# Medication Knowledge of Patients with Parkinson's Disease: Strengths and Gaps

**DOI:** 10.1002/mdc3.70466

**Published:** 2025-12-09

**Authors:** Stephan Greten, Sarana Ulaganathan, Johannes Heck, Lan Ye, Matthias Höllerhage, Christoph Schrader, Corinna Ziemann, Olaf Krause, Florian Wegner, Martin Klietz

**Affiliations:** ^1^ Department of Neurology Hannover Medical School Hannover Germany; ^2^ Institute for Clinical Pharmacology, Hannover Medical School Hannover Germany; ^3^ Center for Medicine of the Elderly, DIAKOVERE Henriettenstift and Department of General Medicine and Palliative Care, Hannover Medical School Hannover Germany; ^4^ Hospital DIAKOVERE Henriettenstift, Center for Geriatric Medicine Hannover Germany

**Keywords:** adherence, device‐aided treatment options, medication knowledge, Parkinson's disease

## Abstract

**Background:**

Effective drug treatment of motor and non‐motor symptoms in Parkinson's disease (PD) often requires the administration of several anti‐Parkinson drugs in complex treatment regimens. The successful autonomous application of drugs places high demands on therapy adherence, which in turn relies on sufficient medication knowledge. However, limited information exists regarding the medication knowledge among PD patients.

**Objectives:**

The aim of this study was to determine the knowledge of PD patients regarding drug treatment, device‐aided therapies and to explore potential factors influencing this knowledge.

**Methods:**

The study was conducted as a prospective, cross‐sectional, interview‐based investigation, enrolling 70 patients. Knowledge on medication was standardized according to the number of drugs taken and expressed as a medication knowledge quotient (MKQ).

**Results:**

The median MKQ was reasonably high at 4.9 (IQR 3.5–5.9), without influence of sex and disease duration. Increasing age (*p* = 0.003), decreasing cognitive function (*p* < 0.001), low educational attainment (*p* < 0.001) and dispensing responsibility for drug provision (*p* < 0.001) negatively influenced MKQ. Knowledge regarding anti‐Parkinson's drugs (ATC N04) was significantly higher than knowledge of other drug classes (*p* < 0.001). In contrast, knowledge of device‐aided treatment options was poor.

**Conclusion:**

PD patients displayed a high level of medication knowledge, particularly regarding anti‐Parkinson's drugs. Age and the accompanying cognitive impairment had a negative impact on medication knowledge. By contrast, knowledge of the key features of device‐aided treatment options requires considerable improvement.

The successful treatment of chronic disorders like Parkinson's disease (PD) relies, among other things, on patient‐specific factors such as adherence to drug recommendations.^1^, ^2^ Poor adherence may not only lead to a direct deterioration of PD symptoms and a corresponding decline in quality of life, but also indirectly generate significant costs for the healthcare system.^3^ A variety of causes have been identified for non‐adherence to drug regimens. The first factor includes structural elements such as access to the healthcare system, availability of drugs, and the patient‐physician relationship.^4^ Drug‐related issues are the second factor to consider, namely the occurrence of adverse effects, as well as the number and frequency of drug administration.^4^ Ultimately, patient‐specific factors affect medication adherence. Along with education and cognitive function, a key element is the knowledge about their disease and their own medication.^4^, ^5^, ^6^

Among chronic diseases, PD certainly occupies a special status with regard to the difficulty of therapy adherence. The treatment of disease‐specific symptoms often requires the administration of several anti‐Parkinson's drugs at numerous time points throughout the day.^1^ In addition, the common comorbidities of the mostly elderly PD patients usually necessitate the prescription of additional drugs.^7^, ^8^, ^9^ Overall, this leads to a high frequency of polypharmacy (administration of ≥5 drugs) and, as a result, an exceptionally complex drug therapy compared to other diseases.^7^, ^10^ To make medication adherence even more challenging, many PD patients regularly develop cognitive impairments, eventually leading to dementia, during the course of the disease.^11^

Previous studies in older patients have already demonstrated that adherence can be improved through solid patient‐physician communication and tailored medication education.^12^, ^13^ Prior to implementing such targeted enhancements to medication knowledge, it would be beneficial to determine specific knowledge deficits. So far, no dedicated studies on this subject have been conducted in PD patients.

Therefore, this prospective interview‐based, cross‐sectional study aimed to systematically assess medication knowledge in PD patients for the first time. Besides knowledge about common anti‐Parkinson's drugs, the study explored the knowledge about device‐aided advanced therapies. Lastly, relationships between medication knowledge and therapy adherence as well as outcomes in activities of daily living were established. For this purpose, the study utilized a questionnaire that had already been established in a study with older patients and used the domains required for the study objectives.^14^

## Methods

### Participants

This study was designed as a prospective, cross‐sectional, interview‐based investigation. The inclusion criteria for participants were a clinical diagnosis of probable PD and a time since diagnosis duration of at least 1 year.^15^, ^16^ The latter criterion was applied to ensure sufficient time for PD patients to familiarize with or be informed about the disease and possible drug treatment options. Subsequently, the potential participants were screened for cognitive impairment using the Montreal Cognitive Assessment (MoCA).^17^ Considering that cognitive impairment might have a negative impact on medication knowledge, patients with a score of 22 or lower in MoCA were excluded from participating in the study. The cut‐off was chosen according to the MoCA validation study.^18^ All enrolled subjects were informed in detail about the objectives of the study and signed a written informed consent. Between October 2022 and February 2024, a total of 117 PD patients were screened during either inpatient or outpatient treatment at the Department for Neurology at the Hannover Medical School and patients from regional patient groups. 37 patients did not meet the inclusion criteria (five patients had a disease duration of less than 1 year, 32 patients revealed cognitive impairment). A further 10 patients did not participate for other reasons (language difficulties, no interest) or quit the survey due to time constraints. Therefore, 70 PD patients were finally recruited.

### Assessments

The participating patients received no financial compensation. Each interview lasted between one and a half hours per patient.

Alongside the basic demographic characteristics (age, sex, disease duration), information on the individual health care structure, for example the frequency of outpatient neurological consultations, and on drug safety issues were obtained, for example the responsibility for regular drug interaction checks.

The centerpiece of the interview was a survey of medication knowledge using a questionnaire specifically developed for this purpose. The questionnaire was created by Krause et al for older patients and tested on a large cohort of geriatric patients.^14^ For this study, the questionnaire was modified and expanded with issues concerning PD patients, namely a section on non‐oral advanced therapies for PD.

The survey began with a self‐assessment of the patient's individual knowledge of medication, using a 5‐point Likert scale from 1 = “very low” to 5 = “very high.” Subsequently, questions were answered regarding knowledge of medication‐related information, including name of the medication, indication, dosage, frequency of use and potential adverse effects. These medication‐related aspects were assessed individually for each drug listed in the patients’ medication plans. Scoring was based on the work of Krause et al, with one point given in the domains “drug name” and “frequency of use,” and up to two points in the domains “indication” and “dosage.”^14^ The points achieved for each drug were summed to generate a medication knowledge score ranging from 0 to 6, with higher scores indicating better knowledge of the medication. To account for differences in the number of medications taken by each patient, the total medication knowledge score was divided by the total number of medications taken. The result was the patient's final average medication knowledge score, referred to here as the medication knowledge quotient (MKQ). Furthermore, the patients’ knowledge of adverse drug reactions (ADR) was queried and evaluated. Finally, the patients’ perception of the daily number of drugs administered was rated using a five‐point Likert scale from 1 = “not enough” to 5 = “too much.”

In the additional section on PD‐specific aspects, the following topics were explored: mentioning of device‐aided treatment options, their detailed specifications and, finally, their criteria for exclusion and suitability. Responses were scored from 0 = “no knowledge,” 1 = “low knowledge” to 2 = “high knowledge.” Examples of patient answers and corresponding ratings are provided in Supplementary Table S1.

The MDS‐Unified Parkinson's Disease Rating Scale part II (MDS‐UPDRS‐II) was performed in all participants to assess the potential influence of medication knowledge on activities of daily living (ADL).^19^ The long‐established tool evaluates motor limitations in daily life using a self‐assessment. Based on the work of Martínez‐Martín et al, patients with an MDS‐UPDRS‐II score of 0 to 12 were considered to have mild, patients with a score of 13 to 29 moderate, and patients with a score of 30 or more severe limitations in ADL.^20^

Lastly, the Stendal Adherence Medication Score (SAMS) questionnaire was conducted in all subjects to evaluate the potential impact of medication knowledge on therapy adherence.^21^, ^22^ The SAMS questionnaire comprises 18 questions with responses rated on a 5‐point Likert scale. The cumulative scale ranges from 0 to 72, with 0 indicating full compliance and 72 indicating complete non‐compliance. Individual medication adherence was categorized as fully adherent (SAMS = 0), moderately non‐adherent (SAMS 1–10), or non‐adherent (SAMS >10) according to Prell et al.^23^ Moreover, a principal components analysis by Prell et al revealed that the individual items of the SAMS could be assigned to three different clusters: modifications (cluster1: items 8–13, 17), lack of knowledge (cluster 2: items 1–3, 5) and forgetfulness (cluster 3: items 6, 14–16).^23^ Besides medication adherence parameters, the SAMS was utilized to obtain additional demographic data, particularly education level and responsibility for providing medication.

### Statistics

All statistical analyses were performed with IBM SPSS Statistics 30.0.0 (Armonk, New York, USA) and GraphPad PRISM 9 (San Diego, CA, USA). Drugs were classified according to the Anatomical Therapeutic Chemical (ATC) Classification for Germany, version 2022.^24^ For statistical analyses, first‐level ATC codes were used.

Quantitative variables are displayed as mean with standard deviation or, due to the skewness of the distribution, medians with interquartile ranges (IQRs). Shapiro–Wilk test was performed to test quantitative variables for normal distribution. Since none of the evaluated variables were normally distributed, Mann–Whitney U or Kruskal‐Wallis test was used for the analyses. For the comparisons of patient groups of different ages and disease duration, the patient cohort was divided according to the median in order to compare groups of equal sample size. The median age was 70 years and the median duration of illness was 8 years. Categorical variables are reported as absolute and relative frequencies. Chi‐squared test was performed to compare proportions for categorical variables. Spearman rank correlation coefficient was used to measure linear correlation between SAMS score and MKQ.

## Results

### Patients’ Characteristics

Data of 70 PD patients are displayed in Table 1. The mean age of the study population was 67.3 ± 11.0 years, with 44.3% of patients being female. The disease duration was 9.3 ± 6.7 years. Nineteen (27.1%) patients were treated with device‐aided advanced therapy, the majority undergoing deep brain stimulation (*n* = 11, 15.7%) and levodopa/carbidopa intestinal administration (*n* = 7, 10%). Almost all patients (66, 94.3%) consulted a neurologist more than once a year. The patients took a median of 7 (IQR 4–9) medications. 79.5% ± 26.3% of the drugs listed in the medication plan were spontaneously recalled by patients.

**TABLE 1:** PD patients’ characteristics

	Patients (*n* = 70)
Sex	
Female, *n* (%)	31 (44.3)
Male, *n* (%)	39 (55.7)
Age, mean ± SD (years)	67.3 ± 11.0
Disease duration, mean ± SD (years)	9.3 ± 6.7
Device‐aided advanced therapies, *n* (%)	
None	51 (72.9)
Deep brain stimulation	11 (15.7)
Apomorphine	1 (1.4)
Duodopa	7 (10)
Neurological consultation, *n* (%)	
None (not treated by a neurologist)	0 (0)
Once a year	4 (5.7)
More than once a year	66 (94.3)
Opinion about number of drugs, *n* (%)	
Too few	1 (1.4)
Rather too few	0 (0)
Appropriate	48 (68.6)
Rather too many	9 (12.9)
Too many	12 (17.1)
Number of drugs taken, median (IQR)	7 (4–9)
Percentage of drugs recalled, mean ± SD	79.5 ± 26.3
MKQ, median (IQR)	4.9 (3.5–5.9)

Abbreviations: IQR, interquartile range; MKQ, medication knowledge quotient; PD, Parkinson's disease; SD, standard deviation.

The majority of patients (*n* = 48, 68.6%) considered the number of prescribed medications to be “adequate” and rated their individual knowledge of medications as “sufficient” (*n* = 25, 35.7%) or “good” (*n* = 28, 40%). The greatest contribution to knowledge about medications was provided either by neurologists (*n* = 26, 37.1%) or the internet (*n* = 17, 24.3%). General practitioners (*n* = 24, 34.3%) were designated as the main coordinator of treatment prior to neurologists (*n* = 18, 25.7%) and others, medication checks mostly fell to the neurologists (*n* = 22, 31.4%) or pharmacists (*n* = 19, 27.1%), according to the patients.

### Medication Knowledge Quotient (MKQ)

**Figure 1:**
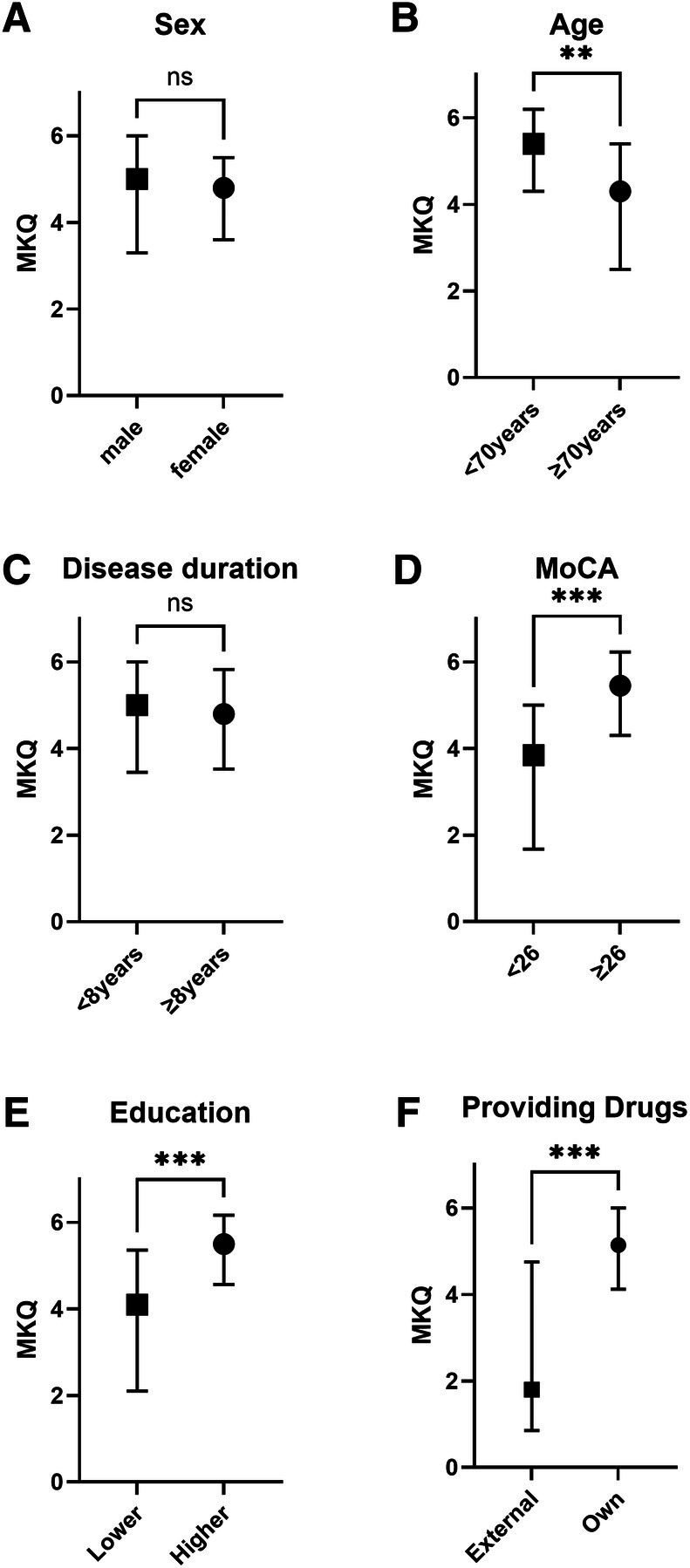
Medication knowledge quotient in selected PD patient subgroups. The figure displays the medication knowledge quotient among subgroups of the investigated PD patient cohort with different sex (A; *n* = 39 male patients; *n* = 31 female patients), age (B; *n* = 35 patients <70 years; *n* = 35 patients ≥70 years), disease duration (C; *n* = 36 patients with a disease duration <8 years; *n* = 34 patients with a disease duration ≥8 years), cognitive function (D; *n* = 26 patients with a MoCA <26; *n* = 44 patients with a MoCA ≥26), education level (E, *n* = 37 patients with a high school/university degree (higher); *n* = 33 with no high school/university degree (lower) and responsibility for providing drugs (F, *n* = 57 patients with own provision; *n* = 13 patients with external provision). ** *p* < 0.01, *** *p* < 0.001, ns non‐significant, Mann–Whitney *U* test. Abbreviations: MKQ, medication knowledge quotient; MoCA, Montreal Cognitive Assessment.

The median MKQ in the study cohort was 4.9 (IQR 3.5–5.9). Certain subgroups displayed no significant differences in the MKQ. Patients of different sexes (female, *n* = 31, median = 4.8 vs. male, n = 39, median = 5; *p* = 0.615, Fig. 1A) and durations of illness (disease duration <8 year, *n* = 36, median = 5 vs. disease duration ≥8 year, *n* = 34, median = 4.8, *p* = 0.694, Fig. 1C) showed comparable scores in MKQ. Age (patients <70 years, *n* = 35, median = 5.4 vs. patients ≥70 years, *n* = 35, median = 4.3, *p* = 0.003, Fig. 1B), cognition (MoCA <26, *n* = 26, median = 3.9 vs. MoCA ≥26, *n* = 44, median = 5.5, *p* < 0.001, Fig. 1D) and education level (high school/university degree (higher), *n* = 37, median = 5.5 vs. no high school/university degree (lower), *n* = 33, median = 4.1, *p* < 0.001, Figure 1E) and responsibility for providing drugs (own provision, *n* = 57, median = 5.1 vs. external provision, *n* = 13, median = 1.8, *p* < 0.001, Fig. 1F) were the identified factors to impact medication knowledge significantly.

The median UPDRS‐II score for patients was 16 (IQR 10–25), indicating a moderate impairment of activities of daily living. No significant correlation between the MKQ and the UPDRS II could be detected (Spearman's *ρ* = −0.197, *p* = 0.102). Yet patients with mild impairment took significantly fewer drugs than patients with moderate impairment as measured by UPDRS II (mild, median = 5 vs. moderate, median = 8 vs. severe, median = 10, *p* = 0.035).

With a median score of 19 (IQR 17–21) in the SAMS, the patient cohort exhibited clinically significant non‐adherence. The medication adherence measured by SAMS did not correlate with the number of administered drugs or the MKQ (number of drugs, Spearman's *ρ* = 0.208, *p* = 0.085; MKQ, Spearman's *ρ* = −0.182, *p* = 0.132). In addition, SAMS clusters were correlated with the medication knowledge data collected. The results showed that cluster 2 (“lack of knowledge”) of SAMS had a significant negative correlation with the number of medications recalled and the MKQ (number of drugs recalled, Spearman's *ρ* = −0.302, *p* = 0.011; MKQ, Spearman's *ρ* = −0.406, *p* < 0.001).

Knowledge about ADR of each drug was not considered in the calculation of the MKQ. Nevertheless, knowledge about ADR, standardized according to the number of administered drugs, correlated significantly with the MKQ (Spearman's *ρ* = 0.605, *p* < 0.001).

Finally, differences in the knowledge of various drug classes in the ATC classification were analyzed. The knowledge of anti‐Parkinson's drugs (median knowledge per drug = 6, ATC N04) was significantly higher than of cardiovascular drugs (median knowledge per drug = 5, ATC C; *p* < 0.001), alimentary tract and metabolism drugs (median knowledge per drug = 5, ATC A; *p* < 0.001), and other drugs (median knowledge per drug = 5, *p* = 0.002).

### Device‐Aided Advanced Therapy Knowledge

Knowledge regarding device‐aided advanced therapies was generally poor. Thus, 58 (82.8%) patients had no (*n* = 5, 7.1%) or low (*n* = 53, 75.7%) knowledge about available options, 64 (91.5%) patients had no (*n* = 16, 22.9%) or low (*n* = 48, 68.6%) knowledge of the therapy procedures, and 67 (95.7%) patients had no (*n* = 47, 67.1%) or low (*n* = 20, 28.6%) knowledge of the inclusion and exclusion criteria for the different therapies. Patients receiving non‐oral advanced therapies demonstrated better knowledge on therapy procedures (*p* = 0.036) and inclusion/exclusion criteria (*p* = 0.004).

**Figure 2:**
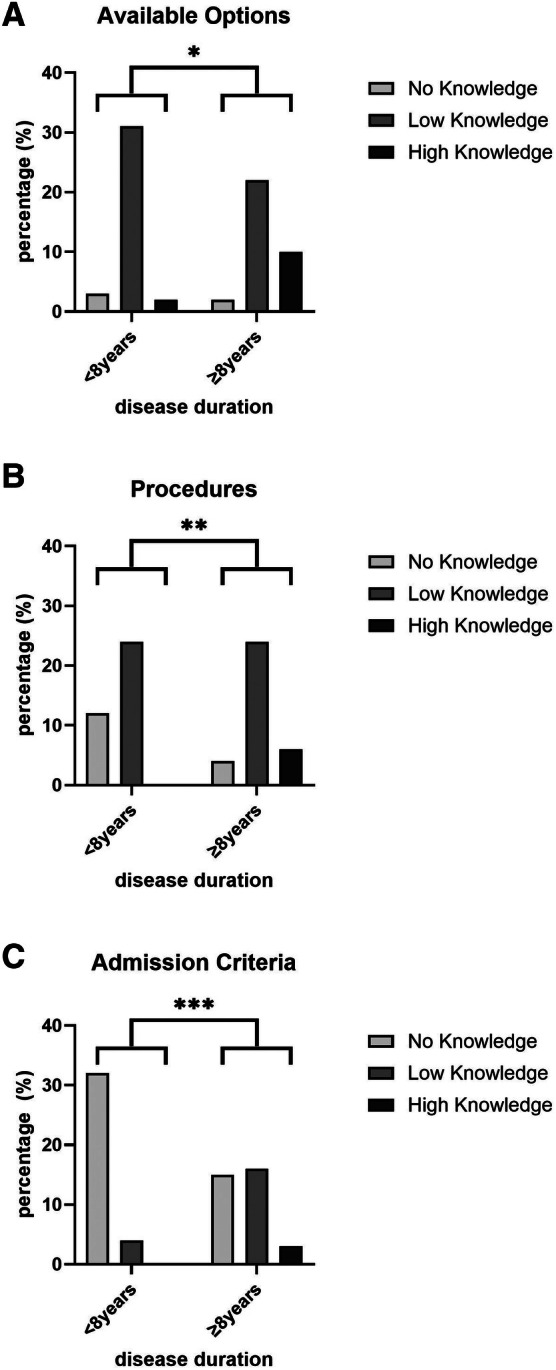
Knowledge of advanced treatment procedures in PD patients of different disease duration. The figure displays the knowledge of PD patients regarding advanced treatment procedures, namely the knowledge of the available options (A), the execution of the procedures (B) and the inclusion/ exclusion criteria (C). The frequencies of no, low or high knowledge in PD patients with different disease durations (*n* = 36 patients with a disease duration <8 years; *n* = 34 patients with a disease duration ≥8 years) for the particular domains are demonstrated. *p < 0.05, **p < 0.01, ***p < 0.001, Chi‐squared test.

In contrast to overall medication knowledge, neither age nor cognition exerted a significant negative impact on the knowledge of advanced treatment procedures. However, Parkinson's patients with a disease duration of at least 8 years performed better in this field (Fig. 2). Long‐term PD patients demonstrated greater knowledge in all domains, namely the available options (high knowledge, 2 (5.6%) vs. 10 (29.4%), *p* = 0.03), procedures (high knowledge, 0 (0%) vs. 6 (17.6%), *p* = 0.007) and selection criteria (high knowledge, 0 (0%) vs. 3 (8.8%), *p* < 0.001).

## Discussion

The data from this cross‐sectional study of PD patients at a university hospital in Germany reveal an overall good level of medication knowledge.^14^ These results are largely consistent with the patients’ self‐perception. According to patients, neurologists have the major influence on medication knowledge education. Age and cognitive function were identified as factors with a negative impact on medication knowledge.

A comparable median MKQ of 5 was found among the subjects in the previous study conducted by Krause and colleagues.^14^ In contrast to the present study, sex‐dependent differences in medication knowledge were observed, namely a reduced MKQ in men.^14^ However, these results may be due to the differing composition of the patient cohort, as the reference study included more women (71%).^14^ Regarding the impact of sex on medication knowledge, results are currently contradictory. In addition to our data, which showed no sex differences on this point, Krause and colleagues showed greater knowledge among women, whereas Okuyan and colleagues reported the opposite.^5^, ^14^

In line with our results, Krause et al and various other studies demonstrated a negative correlation between advancing age and medication knowledge.^5^, ^14^, ^25^, ^26^ The reasons for this association appear to be multidimensional and rooted not only in the patients themselves, but also in their healthcare environment. From a structural perspective, the transfer of responsibility for medication issues to caregivers, staff in nursing homes or physicians, for example, could have a negative impact on medication knowledge. In this regard, the data from the present study did not show any impact of the care setting on medication knowledge (data not shown), but did reveal a positive association of the MKQ with autonomous drug provision. So far, no comparable studies in patients with PD have been conducted focusing on the link between medication knowledge and environmental conditions.

A key patient‐specific variable that adversely affects medication knowledge was the individual cognitive function. By contrast, a greater level of patients’ education led to considerably higher medication knowledge. When exploring the profound cognitive function of PD patients, careful attention should always be paid to these factors, as a connection between the number of education years—hence the level of education—and the risk of cognitive decline seem to exist.^27^, ^28^ At first glance, it seems obvious that cognitive function could have a direct or indirect impact on medication knowledge. To the best of our knowledge, this direct association has not been investigated yet. Kripalani and colleagues found that inadequate literacy skills in older patients led to limited ability to manage medications.^29^ According to Maddigan and colleagues, the latter was defined as the reduced cognitive and functional ability to self‐administer a medication as prescribed.^30^ Furthermore, an indirect association was found in the form of a deterioration in medication adherence as cognitive function declined.^4^, ^31^ Among other things, this was attributed to a reduced knowledge or understanding of the respective illnesses.^4^, ^31^ In our study, PD patients with likely pathological cognitive impairment, defined as MoCA scores of ≤22, were excluded after screening.^18^ Nevertheless, PD patients in the diagnostic gray zone on the MoCA, namely with scores between 23 and 25, were included alongside those with likely normal cognition (MoCA≥26). For the first time, a negative effect of cognitive impairment on the medication knowledge of PD patients has been shown here. This circumstance should be taken into account to improve drug therapy and increase drug safety in PD patients. To further complicate matters, cognitive function may also be compromised by neuropsychiatric disorders that commonly occur in the course of the disease, as well as by certain drugs, particularly such with anticholinergic effects.^32^, ^33^ One conclusion on this basis would be to intensify the drug education and training of cognitively impaired PD patients. A further implication could be to fill in gaps in medication knowledge using digital tools, such as medication reminders on smart phones or watches, as cognitive function deteriorates.^34^ In contrast to the data of other studies, therapy adherence measured via SAMS was independent of patients’ performance in the MoCA (data not shown) and MKQ.^4^, ^5^, ^31^ Ultimately, cognitive function should be assessed at regular intervals in order to counteract the negative influence on medication knowledge at an early stage contributing to the safety and success of the drug therapy.

Despite the fact that the previous study by Krause et al excluded patients taking fewer than five drugs and the patient cohort analyzed was significantly older, the difference regarding the average number of administered drugs per day was negligible (drugs per day, median = 8 in Krause et al vs. median = 7 in the present study).^14^ This observation highlights the complexity of the drug treatment of PD, even without consideration of further complicating factors such as the frequent intake time points throughout the day.

The severity and duration of the disease, as well as the individual manifestation of the symptoms affect the complexity of the necessary drug therapy.^35^, ^36^ Advanced treatment options to serve this particular patient cohort usually comprise device‐aided advanced therapies. These include the administration of the dopamine agonist apomorphine (subcutaneously) or levodopa/carbidopa (subcutaneously/ intestinally) and surgery (deep brain stimulation).^35^, ^36^ Though knowledge about pharmacologic treatment of PD (anti‐Parkinson drugs, ATC N04), was significantly better than for other drug classes, the vast majority of PD patients in our cohort displayed a notable lack of knowledge regarding disease‐specific non‐oral advanced therapies. Marked deficits were found at all levels examined, ie, the available treatments, aspects of treatment procedures, and the inclusion and exclusion criteria for each treatment option.

A Swedish study based on a survey investigated the flow and quality of information regarding medications and advanced therapies in a large cohort of over 3000 PD patients, more than 1000 of whom were classified as having advanced PD.^37^ Access to specialist neurological care initially varied greatly between regions, with only 50% of patients being treated by neurologists in some rural counties. In addition to the structural, country‐specific deficits revealed by the study, the results showed that the treating physicians provided insufficient information about advanced therapies, even though most of patients were interested in these treatments.^37^

Device‐aided therapies represent a great treatment option for advanced PD patients. Accordingly, every PD patient should at least be informed about this therapeutic option during the course of their disease. However, a review by Auffret et al highlighted the difficulties faced by the various stakeholders involved in the treatment situation, demonstrating that this ambition does not correspond to the real world situation.^38^ The data reflect more difficult access to these therapies for women and certain ethnic groups. On the patient side, educational level, insurance coverage, cognitive abilities, and trust in the treating physicians continue to play a decisive role in the decision to initiate a device‐aided therapy. Unfortunately, from a professional point of view, there are still no standardized access routes or selection criteria for advanced PD patients. This, in turn, often combined with a lack of experience with device‐aided therapies, leads to considerable uncertainty among treating colleagues.^38^ Finally, the personal preferences of the practitioners might influence the decision‐making process. The above represents only a partial set of issues that negatively impact the patient‐physician information exchange regarding advanced therapies, thereby reducing access to effective advanced therapies.^38^, ^39^ As a result, PD patients experience high levels of dissatisfaction with their condition and a significant reduction in quality of life.^37^

## Limitations

A certain selection bias limits the transferability of the findings, as the cohort consisted of patients who were regularly seen neurologically at a university hospital and members of a Parkinson's patient group. In this regard, a recent work revealed major differences in patient cohorts between scientific populations and real‐world data.^40^ In order to improve the generalizability of the results, a broader patient sample on a real‐world basis would be necessary. This fact needs to be considered, especially with regard to the better MKQ for anti‐Parkinson's drugs.

Furthermore, this cross‐sectional study provides only a snapshot and does not allow any conclusions about the development of medication knowledge over the course of the disease. This would be interesting not only regarding the impact of medication knowledge on clinical outcome factors (quality of life, (non‐)motor symptoms), but also on therapy adherence.

## Conclusions

The medication knowledge of PD patients, assessed using a specially designed questionnaire, was satisfactory, especially concerning anti‐Parkinson's drugs. Medication knowledge in PD patients was adversely affected by increasing age, impaired cognitive function, lower education level and dispensing responsibility for drug provision. Therefore, cognition should be regularly explored thoroughly to avoid negative effects on medication knowledge and, consequently, on clinical outcome and adherence. Moreover, the patient's environment, namely the caregivers or the facility, should always be integrated into the drug treatment regimen.

Even in this cohort, which was predominantly treated in a specialized outpatient clinic for movement disorders, knowledge of the key characteristics of device‐aided advanced treatment options left considerable room for improvement. Thus, these findings highlight the need for standardized and broader access strategies, as well as earlier educational efforts about advanced therapy options. Having sufficient knowledge about their drugs and treatment options is crucial for PD patients—not only for therapy adherence and drug safety, but also to improve clinical outcomes.

## Author Roles

(1) Research project: A. Conception, B. Organization, C. Execution; (2) Statistical Analysis: A. Design, B. Execution, C. Review and Critique; (3) Manuscript Preparation: A. Writing of the first draft, B. Review and Critique.

S.G.: 1A, 1B, 1C, 2A, 2B, 3A.

S.U.: 1B, 1C, 2C, 3A.

J.H.: 2C, 3C.

L.Y.: 2C, 3C.

M.H.: 1B, 2C, 3C.

C.S.: 1B, 2C, 3C.

C.Z.: 2C, 3C.

O.K.: 2C, 3C.

F.W.: 2C, 3C.

M.K.: 1A, 1B, 2A, 2C, 3A.

## Declarations

**Funding Sources and Conflict of Interest:** This study was funded by a Grant of the German Parkinson's disease association to MK und JH. SG was supported by PRACTIS‐Clinician Scientist Program of Hannover Medical School, funded by the German Research Foundation (DFG, ME 3696/3–1). There were no conflicts of interest relevant to this study to declare.

**Financial Disclosures for the previous 12 months:** MK serves as consultant for Abbvie and Stada; received honoraria for scientific presentations from Abbvie, Ever und Bial. MK was funded by the German Parkinson's disease association and MHH plus foundation (Hannover, Germany) and the Petermax Müller foundation (Hannover, Germany).

**Ethical Compliance Statement:** Ethics committee: Hannover Medical School, Carl‐Neuberg‐Straße 1, 30,625 Hannover, Lower Saxony, Germany, ethikkommission@mh-hannover.de, +49–511–532‐3443/−9812. Ethics vote number: No. 10274_BO_K_2022; date of approval: March 11, 2022. First amendment: August 4, 2022. All patients or legal caregivers provided written informed consent. We confirm that we have read the Journal's position on issues involved in ethical publication and affirm that this work is consistent with those guidelines.

## Supporting information


**TABLE S1** Examples of patient responses and corresponding ratings according to the study questionnaire adapted from Krause et al^14^ ATC, Anatomical Therapeutic Chemical (ATC) Classification; CSAI, continuous subcutaneous apomorphine infusion; DBS, Deep brain stimulation; LCIG, intrajejunal levodopa/carbidopa pump; n.a., not applicable.

## Data Availability

The data supporting the findings of this study are available from the corresponding author Dr. Stephan Greten upon reasonable request.
