# Supplementary material for: Medication Knowledge of Patients with Parkinson's Disease: Strengths and Gaps
Source: Mov Disord Clin Pract. 2025 Dec 9;13(6):1427–34. doi: 10.1002/mdc3.70466 (PMC13307460; doi:10.1002/mdc3.70466)
Supplement: Supplementary file 1 — TABLE S1 Examples of patient responses and corresponding ratings according to the study questionnaire adapted from Krause et al 14 ATC, Anatomical Therapeutic Chemical (ATC) Classification; CSAI, continuous subcutaneous apomorphine infusion; DBS, Deep brain stimulation; LCIG, intrajejunal levodopa/carbidopa pump; n.a., not applicable. [file MDC3-13-1427-s001.docx]

**Supplement**

Table 1. Examples of patient responses and corresponding ratings according to the study questionnaire adapted from Krause et al. (1)

| **Medication related topic** | **Description of answer qualities for 0, 1, or 2 points, and examples of patient answers for the respective categories** | | |
| --- | --- | --- | --- |
|  | **0 points** | **1 point** | **2 points** |
| **Drug name** | The name of the drug is not known by the patient. | The drug can be named correctly by the patient (both generic names and brand names are accepted). Minor errors (e.g., wrong pronunciation, omitted or transposed syllables) are tolerated. | n.a. |
| Antidepressants (ATC N06A) | Drug name not known. | Duloxetine: Cymbalta® | n.a. |
| Antihypertensives (ATC C02) |  | Ramipril: Delix® | n.a. |
| Anti-Parkinson drugs (ATC N04) |  | Levodopa/Benserazide: Madopar® | n.a. |
| Oral anticoagulants (ATC B01A) |  | Apixaban: Eliquis® | n.a. |
| **Indication** | The indication for the drug is not known by the patient. | The indication for the drug can be attributed to an organ (system) by the patient. | The indication for the drug can be stated precisely by the patient |
| Antidepressants (ATC N06A) | Indication not known/ Indication is mistaken | “for my mood ” | “Depression”; “anxiety disorder” |
| Antihypertensives (ATC C02) |  | “For my heart”; “for my circulation” | “Against high blood pressure” |
| Anti-Parkinson drugs (ATC N04) |  | “To walk better”; “for better movement”; “against my tremor” | “Against my Parkinson’s disorder” |
| Oral anticoagulants (ATC B01A) |  | “For blood thinning” | “For blood thinning to prevent a stroke”; “thrombosis”; “atrial fibrillation” |
| **Dosage** | The dosage of the drug is not known by the patient. | The dosage is known semiquantitatively by the patient, that is, in shares of one tablet. | The dosage is known quantitatively by the patient. Answers without precise units of measurement (e.g., gram, milligram, etc.) are tolerated. |
| Antidepressants (ATC N06A) | Dosage not known | ½ tablet; ¼ tablet; 1 tablet | Duloxetine 30 mg |
| Antihypertensives (ATC C02) |  |  | Ramipril 5 mg |
| Anti-Parkinson drugs (ATC N04) |  |  | Levodopa/Benserazide 100 mg |
| Oral anticoagulants (ATC B01A) |  |  | Apixaban 5 mg |
| **Frequency of application** | The frequency of application of the drug is not known by the patient. | The frequency of application is known by the patient. | n.a. |
| Antidepressants (ATC N06A) | Frequency of application not known | Once daily; twice daily; up to six times daily; “1-0-1”; “in the morning and evening” | n.a. |
| Antihypertensives (ATC C02) |  |  |  |
| Anti-Parkinson drugs (ATC N04) |  |  |  |
| Oral anticoagulants (ATC B01A) |  |  |  |
| **Device-aided therapy related topic** | **Description of answer qualities for 0, 1, or 2 points, and examples of patient answers for the respective categories** | | |
|  | **0 points** | **1 point** | **2 points** |
| **Available treatment options** | Treatment options are not known to the patient. | Patients have limited knowledge of treatment options and can only name one. | The patient has a good knowledge of treatment options; all options can be named. |
|  | Treatment options not known. | “I have read about it or heard about it.”; “The brain stimulator.” | “Deep brain stimulation (DBS) and intrajejunal levodopa/carbidopa pump (LCIG) and continuous subcutaneous apomorphine infusion (CSAI).” |
| **Therapy procedure** | Therapy procedure is not known to the patient. | Some aspects of the different treatment options can be named by the patient. | Detailed aspects of the treatment procedures are provided by the patient. |
|  | Therapy procedure not known. | “LCIG: passed through a tube into the abdomen.”; “DBS: implants are placed in the brain.” | “LCIG is a constant application of levodopa/carbidopa via an access to the jejunum (PEJ).”; “Deep brain stimulation involves inserting electrodes into parts of the basal ganglia/thalamus.” |
| **Inclusion and exclusion criteria** | Criteria are not known to the patient. | Criteria for some treatments can be named by the patient. | Detailed criteria for most treatments can be named by the patient. |
|  | Criteria not known. | “I think LCIG can be used in advanced stages of the PD.”; “DBS is not possible for patients with PD dementia.” | “LCIG is particularly useful in PD patients with uncontrollable motor fluctuations. The therapy is not suitable for patients in whom PEJ is not applicable.” |

Abbreviations: ATC, Anatomical Therapeutic Chemical (ATC) Classification; CSAI, continuous subcutaneous apomorphine infusion; DBS, Deep brain stimulation; LCIG, intrajejunal levodopa/carbidopa pump; n.a., not applicable.

**References**

1. Krause O, Ziemann CT, Schulze Westhoff M, Schröder S, Krichevsky B, Greten S, et al. What do older patients know about their medication? A cross-sectional, interview-based pilot study. Eur J Clin Pharmacol. 2023 Oct;79(10):1365–74.
